# Predictors of adverse neonatal outcomes in low-risk nulliparous women at term with meconium-stained amniotic fluid

**DOI:** 10.1007/s00404-026-08394-3

**Published:** 2026-03-27

**Authors:** Marwan Odeh, Maya Frank Wolf, Shira Rosen, Lior Lowenstein, Inshirah Sgayer

**Affiliations:** 1 Department of Obstetrics and Gynecology, Galilee Medical Center, Nahariya, Israel; 2 Azrieli Faculty of Medicine, Bar Ilan University, Safed, Israel

**Keywords:** Meconium, Perinatal outcome, Nulliparity, Low-risk pregnancy

## Abstract

**Objectives:**

Meconium-stained amniotic fluid (MSAF) has been linked to adverse neonatal outcomes, but most evidence comes from heterogeneous cohorts that included multiparous and high-risk women. Nulliparous women without comorbidities are generally considered low risk, yet their outcomes in the presence of MSAF remain less well defined. This study aimed to identify predictors of adverse neonatal outcomes in low-risk nulliparous women at term with MSAF.

**Design:**

A retrospective cohort study.

**Patients:**

Seven hundred sixty low-risk nulliparous women with documented MSAF at term delivery between March 2020 and July 2024.

**Setting:**

A tertiary, university-affiliated medical center.

**Methods:**

Obstetric and neonatal data were extracted from electronic medical records. Maternal, intrapartum, and neonatal characteristics were compared between women with and without an adverse neonatal outcome. The latter was defined as the presence of at least one of the following: a 5-min Apgar score < 7, umbilical artery pH < 7.15, admission to the neonatal intensive care unit, the need for invasive ventilation, meconium aspiration syndrome, or neonatal death. Multivariable logistic regression was performed to identify independent predictors of adverse outcome.

**Results:**

Among women with adverse neonatal outcomes (59, 7.8%) compared to those without adverse outcomes, the mean delivery was earlier (39.3 ± 2.5 vs 40.0 ± 1.0 weeks, *p* < 0.001) and the mean birthweight lower (3171.8 ± 698.8 g vs 3328.7 ± 413.2 g, *p* = 0.009). Prolonged rupture of membranes > 18 h was more common (11.9% vs 3.0%, *p* = 0.004), as was chorioamnionitis (22.0% vs 10.6%, *p* = 0.012). Meconium thickness was greater (*p* = 0.049). In multivariable logistic regression, early term delivery (adjusted odds ratio (aOR) 3.63, 95% CI 1.76–7.51), prolonged rupture of membranes > 18 h (aOR 4.27, 95% CI 1.93–9.47), moderate meconium (aOR 2.46, 95% CI 1.29–4.69), thick meconium (aOR 4.67, 95% CI 2.00–10.91), and chorioamnionitis (aOR 2.74, 95% CI 1.52–4.93) were independent predictors of an adverse neonatal outcome.

**Limitations:**

The retrospective single-center design and limited number of adverse neonatal events may restrict generalizability and preclude assessing risks for specific morbidities.

**Conclusions:**

Among low-risk nulliparous women with MSAF, early term delivery, prolonged rupture of membranes, greater meconium thickness, and chorioamnionitis were associated with adverse neonatal outcomes. These factors may assist in predicting risk and guiding intrapartum management, though validation in larger prospective studies is needed.

## What does this study add to clinical work

**Table:** 

Among otherwise low-risk nulliparous women with meconium-stained amniotic fluid, the presence of thick meconium, prolonged rupture of membrane, early term delivery, or chorioamnionitis may identify pregnancies at increased risk of neonatal morbidity and support closer intrapartum monitoring.	

## Introduction

Meconium, a thick dark-green material composed of intestinal secretions, mucus, desquamated epithelial cells, lanugo, vernix, and amniotic fluid, develops in the fetal intestine early in gestation and accumulates progressively as pregnancy advances [1]. Meconium-stained amniotic fluid (MSAF) occurs in 4–22% of deliveries and in more than 40% of post-term pregnancies [2].

MSAF may warrant clinical attention given its association with adverse perinatal outcomes. The most serious complication is meconium aspiration syndrome (MAS), which can cause airway obstruction, chemical pneumonitis, pulmonary hypertension, and respiratory failure requiring intensive care [3]. MSAF has also been linked to low Apgar scores, umbilical artery acidosis, hypoxic–ischemic encephalopathy, seizures, intraventricular hemorrhage, neonatal sepsis, and prolonged stay in the neonatal intensive care unit (NICU) [2, 4, 5]. In addition, MSAF is associated with increased rates of operative delivery, particularly cesarean section for non-reassuring fetal heart rate patterns [2, 4–22]. The risk appears to be influenced by meconium consistency: while thin meconium may carry limited clinical impact, thick meconium has been consistently associated with higher rates of respiratory and infectious morbidity [9, 10]. Collectively, these findings highlight MSAF as a marker of increased perinatal risk, warranting close intrapartum surveillance and timely neonatal support.

Despite the above, clarifying the true implications of MSAF in specific populations has been challenging, as most studies have included heterogeneous cohorts. Many included women with maternal comorbidities or obstetric complications [3, 4, 9–11], others excluded those with thick meconium [11], and most combined multiparous with nulliparous women [3–11]. Such heterogeneity makes it challenging to distinguish risks attributable to MSAF from those influenced by maternal or obstetric background. Although nulliparous women are generally considered of low risk, the characteristics of their labor may amplify the consequences of MSAF and contribute to neonatal complications [12, 13]. Studying this group, therefore, provides an opportunity to identify predictors of neonatal morbidity in a population in which adverse outcomes are often least anticipated. In this context, the present study aimed to evaluate predictors of adverse neonatal outcomes among low-risk nulliparous women with MSAF at term. The goal was to refine intrapartum risk assessment and to guide clinical management.

## Methods

This retrospective cohort study was conducted at a tertiary university-affiliated medical center and included low-risk nulliparous women who delivered between March 2020 and July 2024. For the purpose of this study, “low-risk” was defined as nulliparous women at term (37–41 + 6 weeks) without major maternal comorbidities or obstetric complications. Exclusion criteria included chronic hypertension, pregestational or gestational diabetes, amniotic fluid disorders (oligohydramnios or polyhydramnios), post-term pregnancy ≥ 42 weeks, multiple gestation, fetal anomalies, small-for-gestational-age fetuses (birthweight < 10th percentile), and elective cesarean delivery. The study population was classified into two groups according to neonatal outcomes: those who experienced an adverse neonatal outcome and those who did not. An adverse neonatal outcome was defined as the presence of at least one of the following: 5-min Apgar score < 7, umbilical artery pH < 7.15, admission to the NICU, the need for mechanical ventilation, MAS, or neonatal death. Each component of the composite neonatal outcome was also evaluated and reported separately.

Maternal demographic characteristics, labor and delivery variables, and neonatal outcomes were retrieved from electronic medical records. Maternal data included age, group B streptococcus (GBS) carriage status, and gravidity. Labor characteristics comprised gestational age at delivery, induction of labor (defined as any pharmacologic or mechanical method used to initiate labor, including oxytocin administration, cervical ripening with a balloon catheter, or prostaglandin use), epidural anesthesia, the need for amnioinfusion, mode of delivery (vaginal, cesarean, or vacuum), duration of membrane rupture, and the length of the second stage of labor. Meconium characteristics were also recorded, including thickness (thin, moderate, or thick) and the timing of appearance. The classification of meconium thickness was based on routine clinical documentation by the attending obstetric team at the time of delivery. The latter was categorized as primary (present at the time of membrane rupture) or secondary (initially clear fluid that became meconium-stained during labor). According to our institutional protocol, all the women with rupture of membranes > 18 h received intravenous ampicillin prophylaxis. Women who were GBS positive also received ampicillin, and those who developed clinical chorioamnionitis were treated with ampicillin and gentamicin. A diagnosis of clinical chorioamnionitis was established based on documented intrapartum findings in accordance with our department practice. It was defined as a maternal intrapartum fever of ≥ 39.0°C, or a temperature between 38.0°C and 38.9°C accompanied by at least one additional clinical sign suggestive of intrauterine infection, including maternal leukocytosis (> 15,000/mm^3^), purulent cervical discharge, or persistent fetal tachycardia (> 160 beats per minute).

The neonatal outcomes assessed included birthweight, Apgar scores at 1 and 5 min, umbilical artery pH, admission to the NICU, respiratory distress syndrome, transient tachypnea of the newborn, hypoglycemia, intraventricular hemorrhage, sepsis, the need for phototherapy due to hyperbilirubinemia, MAS, neonatal encephalopathy, and neonatal death.

### Statistical analysis

Continuous variables were reported as mean ± standard deviation, or as median (interquartile range), as appropriate. Categorical variables were presented as counts (percentages). Differences between the three gestational age groups were analyzed using one-way ANOVA for normally distributed continuous variables and the Kruskal–Wallis test for non-normally distributed continuous variables. Categorical variables were compared across the three groups using Chi-square or Fisher’s exact test.

A multivariable logistic regression model was performed to identify independent predictors of the composite neonatal outcome. Variables included in the multivariable model were selected based on clinical relevance and statistically significant associations in univariate analysis (*p* < 0.05). Variables entered into the model included the gestational age group, induction of labor, meconium timing (primary vs secondary), meconium thickness, and prolonged rupture of membranes (> 18 h). Adjusted odds ratios (aOR) with 95% confidence intervals (CI) were calculated, and statistical significance was set at *p* < 0.05. A significance threshold of *p* < 0.05 was applied for the multivariable analysis. All the statistical analyses were conducted using SPSS version 26 (IBM Corp., Armonk, NY, USA).

## Results

During the study period, a total of 1012 nulliparous women delivered at term with meconium-stained amniotic fluid. Of these, 112 were excluded due to preexisting maternal conditions or pregnancy-related conditions (e.g., hypertension, diabetes, amniotic fluid disorders, and post-term pregnancies ≥ 42 weeks), 12 due to multiple gestations, 18 due to fetal anomalies, 76 due to small-for-gestational-age fetuses (birthweight < 10th percentile), and 34, because delivery occurred by elective cesarean section. After exclusions, the cohort comprised 760 women with documented meconium-stained amniotic fluid at delivery. Among them, 59 had adverse neonatal outcomes, while 701 did not.

Among women whose neonates experienced adverse outcomes compared to women whose neonates did not, the proportion with prolonged rupture of membranes (> 18 h) was higher (11.9% vs 3.0%, *p* = 0.004). For the former compared to the latter group, the mean gestational age was earlier (39.3 ± 2.5 vs 40.0 ± 1.0 weeks, *p* < 0.001) and the mean birthweight was lower (3171.8 ± 698.8 g vs 3328.7 ± 413.2 g, *p* = 0.009). Moderate meconium thickness was more common in the adverse outcome group (83.1% vs 71.3%). Table 1 shows maternal and labor characteristics of the two study groups. There were no significant differences between the groups in maternal age, the proportion of women with BMI > 35, or the rate of conception via assisted reproductive technology. Ten women (16.9%) in the adverse outcome group delivered at 41–41 + 6 weeks, compared with 118 women (16.8%) in the group without adverse neonatal outcomes (*p* = 1.00).

**Table 1:** Maternal and labor characteristics of the two study groups (with and without neonatal adverse outcomes)

	Neonatal adverse outcome (*N* = 59)	No neonatal adverse outcome (*N* = 701)	*p* value
Maternal age, mean (± SD)	27.5 (± 4.6)	27.0 (± 4.5)	0.364
Gravity, median (IQR)	1 (1–2)	1 (1–2)	0.598
BMI > 35	9 (15.3)	97 (13.8)	0.845
ART	4 (6.8)	45 (6.4)	1.00
GBS carrier, *n* (%)	6/38 (15.8)	102/413 (24.7)	0.242
Meconium thickness			0.049
Thin	5 (8.5)	149 (21.3)	
Moderate	49 (83.1)	500 (71.3)	
Heavy	5 (8.5)	52 (7.4)	
Secondary meconium, *n* (%)	9 (15.3)	53 (7.6)	0.047
Labor induction, *n* (%)	16 (27.1)	161 (23.0)	0.521
Epidural anesthesia, *n* (%)	39 (66.1)	486 (69.3)	0.660
Second stage length	2.4 (0.8–3.0)	1.7 (0.7–2.8)	0.209
Time from ROM to delivery, hours, median (IQR)	5.9 (1.4–10.9)	5.3 (2.5–8.8)	0.648
ROM > 18 h, *n* (%)	7 (11.9)	21 (3.0)	0.004
GA at birth, mean (± SD)	39.3 (± 2.5)	40.0 (± 1.0)	< 0.001
Birthweight, mean (± SD)	3171.8 (± 698.8)	3328.7 (± 413.2)	0.009
Gender, *n* (%)			1.00
Male	31 (52.5)	363 (51.8)	
Female	28 (47.5)	338 (48.2)	

*SD* standard deviation, *IQR* interquartile range, *BMI* body mass index, *ART* artificial reproductive technology, group B streptococcus, *ROM* rupture of membranes, *GA* gestational age

Table 2 presents intrapartum outcomes and delivery characteristics of the two study groups. Among the women whose neonates had versus did not have adverse outcomes, chorioamnionitis was more frequent (22.0% vs 10.6%, *p* = 0.012) and emergency cesarean delivery was more common (37.3% vs 23.3%, *p* = 0.034). Emergency cesarean section for non-progressive labor was more frequent in the adverse outcome group (22.0% vs 6.6%, *p* < 0.001). There was no statistically significant difference in cesarean delivery for non-reassuring fetal heart rate between women with and without adverse neonatal outcomes (33.9% vs 22.5%, *p* = 0.055). Table 3 presents neonatal complications of the two study groups. Hypoglycemia was more frequent among the neonates with adverse outcomes (13.6% vs 0.1%, *p* < 0.001), as was the need for phototherapy (13.6% vs 5.4%, *p* = 0.02). Respiratory morbidity was greater in this group, with higher rates of respiratory distress syndrome (11.9% vs 0%, *p* < 0.001) and transient tachypnea of the newborn (23.7% vs 0.1%, *p* < 0.001). Severe complications were also more common, including intraventricular hemorrhage (3.4% vs 0.1%, *p* = 0.017) and neonatal sepsis (3.4% vs 0%, *p* = 0.006). Necrotizing enterocolitis and neonatal death were not observed in either group.

**Table 2:** Intrapartum outcomes and delivery characteristics of the two study groups

	Neonatal adverse outcome (*N* = 59)	No neonatal adverse outcome (*N* = 701)	*p* value
5-min Apgar score < 7	3 (5.1)	0 (0)	< 0.001
Umbilical artery pH < 7.15	4/12 (33.3)	0/61 (0)	< 0.001
Placental abruption	1 (1.7)	1 (0.1)	0.149
Intrapartum fever	19 (32.2)	87 (12.4)	< 0.001
Chorioamnionitis	13 (22.0)	74 (10.6)	0.012
Mode of delivery			0.034
Normal delivery	490 (69.9)	32 (54.2)	
Vacuum extraction	48 (6.8)	5 (8.5)	
Cesarean delivery	163 (23.3)	22 (37.3)	
*Indications for cesarean delivery*			
Non-progressive labor	13 (22.0)	46 (6.6)	< 0.001
Non-reassuring fetal heart rate	20 (33.9)	158 (22.5)	0.055 (0.038 ^*^one sided)
Placental abruption	1 (1.7)	0 (0)	0.078
Failed labor induction	0 (0)	2 (0.3)	1.00
Cord prolapse	0 (0)	1 (0.1)	1.00

The data are presented as number (%)

**Table 3:** Neonatal complications of the two study groups

	Neonatal adverse outcome (*N* = 59)	No neonatal adverse outcome (*N* = 701)	*p* value
NICU admission	49 (83.1)	0 (0)	< 0.001
Need for ventilation	36 (61.0)	0 (0)	< 0.001
Hypoglycemia	8 (13.6)	1 (0.1)	< 0.001
Need for phototherapy	8 (13.6)	38 (5.4)	0.02
RDS	7 (11.9)	0 (0)	< 0.001
TTN	14 (23.7)	1 (0.1)	< 0.001
NEC	0 (0)	0 (0)	NA
IVH	2 (3.4)	1 (0.1)	0.017
Neonatal sepsis	2 (3.4)	0 (0)	0.006
MAS	12 (20.3)	0 (0)	< 0.001
Neonatal death	0 (0)	0 (0)	NA

The data are presented as number (%)

*NICU* neonatal intensive care unit, *RDS* respiratory distress syndrome, *TTN* transient tachypnea of newborn, *NEC* necrotizing enterocolitis, *IVH* intraventricular hemorrhage, *MAS* meconium aspiration syndrome, *NA* not available

**Fig. 1:**
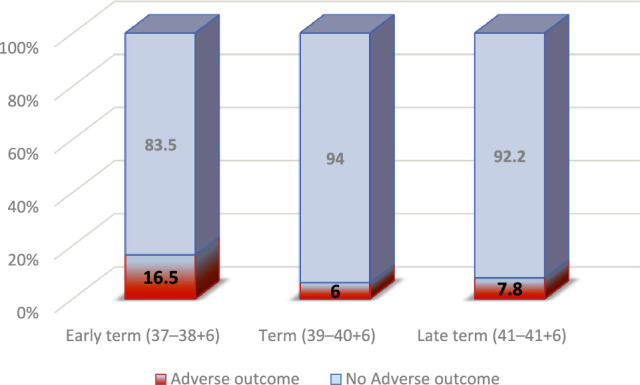
The prevalence of neonatal outcomes according to gestational age at delivery (*p* < 0.001)

**Fig. 2:**
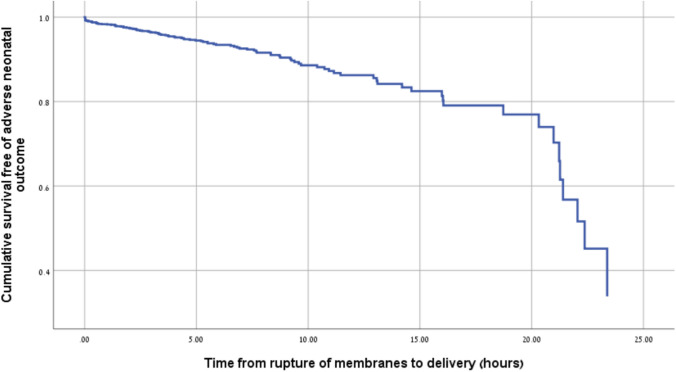
Cumulative survival analysis for adverse neonatal outcomes according to latency after membrane rupture

Multivariable logistic regression analysis identified several variables that were associated with adverse neonatal outcomes (Table 4). Early compared to late-term deliveries (37–38 + 6 weeks) were associated with an increased risk (aOR 3.63, 95% CI 1.76–7.51, *p* < 0.001). Prolonged rupture of membranes > 18 h was associated with an aOR of 4.27 (95% CI 1.93–9.47, *p* < 0.001). Meconium thickness showed a graded effect: moderate meconium was associated with an aOR of 2.46 (95% CI 1.29–4.69, *p* = 0.006) and thick compared to thin meconium with an aOR of 4.67 (95% CI 2.00–10.91, *p* < 0.001). Chorioamnionitis was associated with an aOR of 2.74 (95% CI 1.52–4.93, *p* = 0.001). Secondary meconium had an aOR of 0.57 (95% CI 0.30–1.09, *p* = 0.090) and did not reach statistical significance. Figure 1 presents the distribution of adverse neonatal outcomes across gestational age groups (*p* < 0.001). Figure 2 presents the probability of adverse neonatal outcomes in relation to the duration of membrane rupture.

**Table 4:** Multivariable logistic regression analysis of predictors of adverse neonatal outcome

Variable	*p* value	Adjusted OR	95% CI
GA group ^*^late term (ref.)	< 0.001		
Early term (37–38 + 6)	< 0.001	3.63	1.76−7.51
Term (39–40 + 6)	0.705	0.90	0.53−1.54
ROM > 18 h	< 0.001	4.27	1.93−9.47
Meconium thickness ^*^Light meconium (ref.)	0.002		
Moderate	0.006	2.46	1.29−4.69
Thick	< 0.001	4.67	2.00−10.91
Secondary meconium	0.090	0.57	0.30−1.09
Chorioamnionitis	0.001	2.74	1.52−4.93
Labor induction	0.096	1.52	0.93−2.48

*OR* odds ratio, *CI* confidence interval, *GA* gestational age, *ROM* rupture of membranes

## Discussion

We found that adverse neonatal outcomes occurred in about 8% of low-risk nulliparous women at term with meconium-stained amniotic fluid. Independent predictors of these outcomes included early term delivery, prolonged rupture of membranes, thicker meconium, and intrapartum chorioamnionitis.

Early term delivery (37–38⁶⁄₇ weeks) emerged as a vulnerable period, as evident from the more than threefold greater risk of adverse outcomes compared with late-term births (41–41⁶⁄₇ weeks). Outcomes at 39–40⁶⁄₇ weeks were comparable to late term. Similarly, Hiersch et al., who evaluated low-risk term pregnancies including both nulliparous and multiparous women with meconium-stained amniotic fluid, found that early term births carried the greatest burden of morbidity. For early term compared with full term, the odds were higher of NICU admission, sepsis, jaundice, phototherapy, and low Apgar scores [1]. Although late-term births (41–41⁶⁄₇ weeks) in their cohort were associated with a higher risk of operative delivery and low Apgar scores, the spectrum of complications was narrower than that observed at early term [14]. Taken together, both studies indicate that full-term deliveries (39–40⁶⁄₇ weeks) carry the most favorable prognosis, whereas early term birth in the presence of meconium represents the highest-risk subgroup. Lavie et al. found no differences in their primary composite neonatal outcome, between early term (37–39 weeks, *n* = 239) and late-term (40–42 weeks, *n* = 362) births complicated by MSAF. This composite included a 5-min Apgar score < 7, umbilical artery pH < 7.1, and serious neurologic morbidity (seizures, intraventricular hemorrhage, asphyxia, or ischemic encephalopathy) [10]. Their study included both nulliparous and multiparous women, and both low- and high-risk pregnancies, and excluded those with thick meconium. The authors explained their results by suggesting that meconium passage at term may often represent a benign, physiologic process of gastrointestinal maturation, particularly in later gestation [10]. By contrast, our more homogeneous cohort of low-risk nulliparas demonstrates that gestational age stratifies risk. We hypothesize that the excess morbidity observed in early term births reflects relative fetal immaturity, whereby meconium is more likely to be a marker of intrauterine stress rather than of physiological maturation. It is important to consider whether the association between early term delivery and adverse neonatal outcomes reflects gestational age itself or clinical decision-making (confounding by indication). Early term births may occasionally result from obstetric intervention due to suspected fetal compromise or evolving intrapartum concerns. However, induction of labor was included in the multivariable regression model, and early term delivery remained independently associated with adverse neonatal outcome after adjustment. These findings suggest that the observed association is unlikely to be explained solely by obstetric intervention. Nevertheless, residual confounding from unmeasured clinical factors cannot be entirely excluded.

In our cohort, greater meconium thickness was strongly associated with neonatal morbidity. Specifically, compared with light meconium, moderate meconium doubled the risk of adverse outcomes, while thick meconium increased the risk nearly fivefold. These results extend prior observations. Among nulliparous women of ≥ 41 week gestation, including both low- and high-risk pregnancies, Attali et al. reported that only thick and not thin meconium was associated with neonatal morbidity [15]. In contrast, among both multiparous and high-risk women, Schreiber et al. demonstrated that even thin meconium was linked to intrapartum complications, higher cesarean delivery rates for non-reassuring fetal heart rate, and neonatal morbidities, such as MAS and respiratory distress [11]. By focusing exclusively on low-risk nulliparas across the entire term range, our study provides evidence of a stepwise, graded relation between meconium thickness and neonatal outcome. This pattern is biologically plausible: thin meconium may in part represent physiologic gastrointestinal maturation, whereas increasing thickness is more likely pathological, reflecting possible fetal hypoxia [16]. Thick meconium carries higher concentrations of bile pigments, enzymes, and pro-inflammatory mediators, and has been linked to infection and pulmonary injury [2, 17], which together explain its stronger association with adverse outcomes.

Our analysis demonstrated that when membrane rupture extended beyond 18 h, the presence of meconium-stained amniotic fluid was linked to a higher likelihood of adverse neonatal outcomes. Our findings corroborate a study of 1,631 women with term spontaneous PROM, of whom 536 had MSAF (of whom 263 were nulliparas) and 1,095 had clear amniotic fluid [18]. This heterogeneous cohort spanned parity categories and risk profiles. The authors reported that prolonged exposure to meconium was associated with a stepwise increase in adverse neonatal outcomes. The composite outcome—MAS, neonatal asphyxia, the need for respiratory support, or intracranial hemorrhage—rose from 1.9 to 8.2% when rupture-to-delivery increased from less than 7 to more than 18 h. By contrast, no similar pattern was observed among women with clear amniotic fluid. Maternal outcomes in that study did not differ between MSAF and the clear fluid groups [18]. The authors explained these findings by suggesting that the deleterious effects were specific to meconium itself, whose prolonged presence amplifies neonatal respiratory and neurologic risks. In contrast, rupture alone in the absence of meconium did not carry the same impact [18].

Chorioamnionitis was likewise identified as an independent predictor of adverse neonatal outcomes. While one might expect this association to be mediated by prolonged rupture of membranes, our data suggest otherwise. Rates of clinical chorioamnionitis were comparable between women with rupture < 18 h and those with rupture > 18 h (11.5% vs. 10.7%, *p* = 1.00). This indicates that chorioamnionitis contributes to neonatal morbidity beyond the effect of rupture duration alone. The absence of an association between prolonged rupture of membranes and chorioamnionitis in our cohort may partly reflect our institutional protocol of administering prophylactic intravenous antibiotics after 18 h of membrane rupture, which could have mitigated infection risk. In contrast, Attali et al. reported a progressive rise in chorioamnionitis rates with longer rupture, thus highlighting possible differences in population characteristics or management practices [18]. Whether prophylactic antibiotics should be used in the setting of meconium-stained amniotic fluid has been raised in small studies [19, 20], yet the evidence remains limited. Current data are insufficient to determine whether antibiotics reduce infection-related morbidity in this context. Rather than applying a uniform approach, future research should focus on identifying the women with MSAF who are truly at increased risk for chorioamnionitis and who might benefit from timely antimicrobial therapy.

We also explored the impact of secondary meconium, defined as meconium passage after previously clear amniotic fluid. Although univariate analysis suggested a higher rate of secondary meconium among those with adverse outcomes, this association did not persist in multivariable models. The lack of significance may reflect interactions between multiple predictors or limitations of sample size. Previous studies have suggested that secondary meconium reflects acute intrapartum stress and poses a higher risk than primary meconium [21, 22], as the latter may be related to evolving fetal compromise. However, in our homogeneous cohort, we did not find an independent effect. These findings underscore the complexity of meconium-related morbidity and the need for larger studies to disentangle the contributions of rupture duration, infection, and the timing of meconium passage.

This study provides important insight into predictors of adverse neonatal outcomes in low-risk nulliparous women at term with meconium-stained amniotic fluid. By restricting the cohort to a homogeneous population, we minimized confounding from multiparity and preexisting maternal conditions. Another strength was the relatively large sample size and access to detailed clinical records, which enabled a comprehensive assessment of maternal, intrapartum, and neonatal variables. The application of multivariable regression enabled identifying factors that appeared to act independently. Nonetheless, several limitations should be considered. The retrospective design raises the possibility of selection and information bias. The composite outcome combined complications of differing severity, which precludes interpretating risks for specific morbidities. In addition, the number of adverse events was modest, restricting the number of predictors that could be reliably included in regression models. In addition, the specific indications for induction of labor were not systematically recorded in a structured format within the database, precluding assessment of potential confounding by indication. In addition, detailed data regarding neonatal sepsis evaluation and postnatal antibiotic treatment were not systematically available, limiting further characterization of infection-related neonatal morbidity. The number of adverse events was modest, which may have limited the number of predictors that could be reliably included in the multivariable model and raises the possibility of model overfitting. Therefore, the regression estimates should be interpreted with caution and require validation in larger cohorts. Finally, as this was a single-center study, the findings may not be generalizable to other populations or practice settings.

## Data Availability

All the data analyzed during this study are included in this manuscript. Further enquiries can be directed to the corresponding author.
